# Identifying COVID-19 survivors living with post-traumatic stress disorder through machine learning on Twitter

**DOI:** 10.1038/s41598-024-69687-8

**Published:** 2024-08-14

**Authors:** Anees Baqir, Mubashir Ali, Shaista Jaffar, Hafiz Husnain Raza Sherazi, Mark Lee, Ali Kashif Bashir, Maryam M. Al Dabel

**Affiliations:** 1 Complex Human Behavior Laboratory, Fondazione Bruno Kessler, Trento, Italy; 2 School of Computer Science, University of Birmingham, Birmingham, UK; 3 National Drug and Treatment Center, Dublin, Ireland; 4 School of Computing, Newcastle University, Newcastle Upon Tyne, UK; 5 Department of Computing and Mathematics, Manchester Metropolitan University, Manchester, UK; 6 Woxsen School of Business, Woxsen University, Hyderabad, 502 345 India; 7 Department of Computer Science and Mathematics, Lebanese American University, Beirut, Lebanon; 8 Department of Computer Science and Engineering, College of Computer Science and Engineering, University of Hafr Al Batin, Hafar Al-Batin, Saudi Arabia; 9 Northeastern University, London, UK

**Keywords:** Post-traumatic stress disorder, Computer science

## Abstract

The COVID-19 pandemic has disrupted people’s lives and caused significant economic damage around the world, but its impact on people’s mental health has not been paid due attention by the research community. According to anecdotal data, the pandemic has raised serious concerns related to mental health among the masses. However, no systematic investigations have been conducted previously on mental health monitoring and, in particular, detection of post-traumatic stress disorder (PTSD). The goal of this study is to use classical machine learning approaches to classify tweets into COVID-PTSD positive or negative categories. To this end, we employed various Machine Learning (ML) classifiers, to segregate the psychotic difficulties with the user’s PTSD in the context of COVID-19, including Random Forest Support Vector Machine, Naïve Bayes, and K-Nearest Neighbor. ML models are trained and tested using various combinations of feature selection strategies to get the best possible combination. Based on our experimentation on real-world dataset, we demonstrate our model’s effectiveness to perform classification with an accuracy of 83.29% using Support Vector Machine as classifier and unigram as a feature pattern.

## Introduction

The COVID-19 virus, which rapidly spread across the world since late 2019, was first detected on December 31, 2019. World Health Organization (WHO) declared it a pandemic On March 11, 2020. As of March 13, 2023, the virus has infected people in 216 countries, with over 759.41 million confirmed cases and over 6.87 million confirmed deaths^1^. In response to the pandemic, educational facilities in 190 countries were closed, and many governments issued flight bans and stay-at-home orders, affecting people worldwide^2^.

Prior to the COVID-19 outbreak, an estimated 380 million people worldwide, of all ages, were affected by mental health issues. Previous studies have shown that mental health problems can lead to harmful outcomes such as suicide^3,4^. However, these studies face two major challenges. Firstly, many individuals with mental health problems are hesitant or ashamed to seek help^5^. Secondly, obtaining and analyzing a large sample size of diagnosed individuals can be difficult in psychological research.

Numerous studies have investigated the economic and social impacts of COVID-19^6,7^. In addition, various investigations have revealed that the mental health of people around the world has been greatly affected by the COVID-19 outbreak. These studies have reported higher rates of depression, anxiety, PTSD, and stress symptoms during the pandemic than before^8^. While stay-at-home orders and social distancing measures have been effective in preventing the spread of COVID-19, as suggested by previous research, they can also have negative effects on individuals’ mental health^9–11^.

Social media data can provide valuable insights into physical and mental health concerns. Often, social media users are unaware of changes in their own health^12^. Research has demonstrated that searching for information about certain health problems can reveal early-warning signs of hard-to-detect tumors^13^. Social media platforms have also been utilized to track outbreaks of illness and monitor regional nutrition^14,15^. Predictive screening algorithms have been successful in identifying signals in social media data for a variety of mental health conditions, including addiction^16^, suicide ideation^17^, depression^18–20^, Post-Traumatic Stress Disorder (PTSD)^21^ as well as physical ailments. However, the use of predictive health screening through social media data is still in its early stages, and significant modifications are needed to establish approaches that can effectively supplement health treatment.

### Motivation

Depression is a prevalent mental health condition that affects a wide range of behaviors and communication patterns, making it a major concern among computational social scientists^22–24^. Despite its prevalence, depression is often underdiagnosed, with studies reporting that up to 45% of cases in some major metropolitan regions remain undiagnosed^25^. Post-traumatic stress disorder (PTSD) is less common than depression, but it is often associated with significant levels of depression^26^. Primary-care physicians frequently underdiagnose or undertreat PTSD^27^, highlighting the potential benefits of computational approaches for early screening and diagnosis of depression and PTSD^12^.

Given these concerns, we aim to explore how COVID-19 has impacted people’s mental well-being and determine what percentage of individuals have been affected. Moreover, our objective is to create a machine learning model that can classify individuals as having PTSD or not, based on their exposure to COVID-19, with a high degree of accuracy.

### Research contributions

The present study achieves the following four-fold contributions:

- The dataset of more than 3.96 Million Tweets has been constructed from the users who mentioned on their Twitter timeline that they were COVID positive at some point between March 2020 and November 2021.
- The resulting dataset has been filtered and manually annotated following International statistical Classification of Diseases (ICD)-11^28^ guidelines.
- The proportion of users was quantified being PTSD positive or negative based on the data filtration criteria which gives us a better understanding of users’ posting behavior after they were diagnosed with COVID.
- Finally, a machine learning based classification model has been proposed to effectively classify the tweets of users as either PTSD positive or negative.

Rest of the paper is organized as follows. Section “Post-traumatic stress disorder (PTSD)” discusses the PTSD and its diagnosis along with the guidelines adopted for data filtering and annotation. Section “Literature review” sheds light on the state of the art on the topic while section “Methodology” explains the proposed methodology for the study along with a brief description of our chosen classification algorithms. In section “Data extraction”, we discuss our approaches of data extraction, filtration and annotation along with our findings based on the data. And finally in section “Conclusion”, we conclude our findings and mention our future directions.

## Post-traumatic stress disorder (PTSD)

PTSD is a type of anxiety disorder that can develop in individuals who have experienced a traumatic event, such as a car accident, war, physical, emotional, or sexual abuse, a natural disaster, or any other life-altering experience that impacts their biological or psychological state. The WHO and the American Psychiatric Association (APA) both recognize PTSD as a legitimate condition, and diagnostic criteria are provided in ICD and the Diagnostic and Statistical Manual of Mental Disorders (DSM), as well as related health problems^29^.

PTSD is a conglomerate of symptoms affecting multiple domains and it is described as “the complex somatic, cognitive, affective, and behavioral effects of psychological trauma”^30^. Considering lack of physical symptoms in most cases of PTSD and the stigma attached to mental illness, a lot of times, PTSD is diagnosed in people after months of struggling with it. The fact that there is no blood test or an imaging test that can help diagnose PTSD right away is also a barrier to effective treatment being offered at an earlier stage. Population struggling with PTSD are late to be identified and they mostly come to light when they start to struggle at work, have difficulties in their relationships with others, or become addicted to drugs or alcohol to self-medicate to numb their symptoms. Once the contact is made with a psychiatrist, a thorough history of the traumatic event, the symptoms related to it, and in many cases collateral history is necessary to make the right diagnosis.

A cross-sectional study carried out on nurses exposed to COVID in China found incidence of PTSD to be 16.8%, with highest scores in avoidance symptoms^31^. Our aim in this study is to be able to cut short this lengthy process of diagnosis of PTSD by recognizing those who have had COVID and might be suffering from PTSD using their tweets. By identifying this population and predicting that they might have PTSD, they can be offered proper evaluation and optimal treatment

We acknowledge the complexity of post-traumatic stress disorder (PTSD) and recognize that its diagnosis extends beyond language patterns alone. Factors such as context and personal history are integral to understanding and assessing PTSD. It’s important to emphasize that the severity and impact of the traumatic incident also play a significant role in determining the presence of PTSD symptoms.

PTSD, classified as an anxiety disorder, often emerges following exposure to a traumatic event, which may involve actual or threatened death. We posit that COVID-19 presents a potential trigger for PTSD due to the profound trauma associated with the experience, coupled with the pervasive fear of mortality. Despite potential limitations, our computational analysis serves as a valuable screening tool to identify individuals at risk of developing PTSD. Notably, symptoms such as hyper-vigilance, anxiety, insomnia, flashbacks, and nightmares, consistently observed in our study, correlate with individuals’ encounters with COVID-19. Moreover, various factors, including witnessing severe illness or death, enduring prolonged isolation, fear of contagion, and uncertainty about the future, further contribute to this psychological response. While our study may not fully meet diagnostic criteria, it lays a crucial groundwork for screening populations susceptible to PTSD, thereby facilitating the development of tailored services for timely diagnosis and intervention.

## Literature review

The field of mental health detection has been the focus of numerous studies utilizing various datasets and modeling techniques to develop reliable models for detecting mental health issues. In such study by Joshi et al.^32^, a combination of deep learning and conventional machine learning algorithms was used to detect mental health issues through social media posting and behavioral features. The first stage of classification involved considering 13 behavioral features to classify users, while in the second stage, a behavioral feature called DL_score was created using a word2vec model to classify tweets. The model was trained on nearly 12 million tweets for tweet classification. Their model achieved an accuracy of 89%, with the deep learning feature extraction helping to accurately classify users as normal or non-normal, while also reducing the false positive rate.

During current pandemic, 36.6 million users tweeted almost 41.3 million COVID-19 related tweets in 2020^33^. Based on COVID-19 related tweets, the keywords like ‘corona’, ‘#Corona’, ‘covid19’ etc. tweets are collected from the profile description and tweets of the users to look for the signs of depression. Among 2575 twitter users, 200 are randomly selected from the classified depression set of users and 86% are labeled as positive. Almost 1402 depression users tweeted the tweets that are chosen, posted in three months of time span. Transformer-based models such as BERT, RoBERTa, and XLNet are applied to identify depression users to monitor depression trend during COVID-19.

A study by Sekulic et al.^34^ proposed a Hierarchical Attention Network (HAN) for the detection of mental disorders. This model is comprised of a word sequence encoder, a layer at the word-level attention, a sentence encoder, and a layer at sentence-level attention. Initially, users with a self-reported diagnosis of nine mental disorders were identified, and the model was trained on their posts, which were modeled as sentences. The HAN outperformed baseline models in detecting depression, anxiety, ADHD, and bipolar disorders, but performed inadequately for PTSD, autism, eating disorders, and schizophrenia. With the attention mechanism provided by the HAN, important words or phrases were easily identified and deemed relevant for classification.

The study utilized lists of n-grams derived from tweets of users diagnosed with depression or PTSD, which were used to train a classifier to rank tweets of other users as positive or negative for depression or PTSD^35^. The dataset consisted of tweets from 327 random Twitter users, out of which 246 users reported a PTSD diagnosis and had at least 25 tweets. The tweets related to each condition were randomly selected, and the first eight million words of tweets were used in the training data. The features were selected based on their frequency of occurrence, with n-grams that occurred 50 times more in a single condition being included. This selective approach to feature selection helped to improve the results and provide greater insight into the identification of mental illness via social media posts.

To identify individuals who may be experiencing depression, a group of Twitter users who had self-reported their diagnosis of depression via tweets were selected using the Twitter streaming API^36^, with regular expressions and data acquisition techniques being used over a four-month period. To ensure a balanced dataset, authors selected equal number of positive and negative instances representing depressive and non-depressive tweets respectively from 600 randomly selected users to perform the experiments. Features for emotions were extracted, and strength scores were assigned to create emotion-based features, while time-series analysis was applied, and descriptive statistics were selected as temporal features. The resulting model achieved an accuracy of 87.27% on emotion features alone, outperforming baseline models^18,37,38^. When different temporal features were used, the accuracy was improved with 89.77%, and when both, i.e., emotion and temporal features were combined, the accuracy increased to 91.81%. These findings suggest that basic emotions can be used to identify individuals who may be experiencing depression on Twitter.

To conduct their research, the authors utilized a widely recognized dataset in the fields of computational linguistics and clinical psychology^39^, which comprised of three types of Twitter users: those who self-reported a diagnosis of depression, those who self-reported having PTSD, and a control group of users matched in terms of demographics^40^. The dataset consisted of 3000 tweets, which were manually reviewed to eliminate irrelevant information. The authors then conducted a qualitative analysis to identify instances of misclassification in their approach, discovering that some false positives arose from the use of language that displayed anger or frustration, while other false positives were linked to music, bands, or artists associated with the positive class. The authors emphasized the limitations of using similar machine learning systems and the importance of not relying solely on automated classifiers to determine an individual’s mental health status on social media platforms.

**Table 1:** Comparison of results with previous studies.

Reference	Probelm	Related to covid	Data source	Methodology	Result
^32^	Classification of users with anxiety, bipolar disorder etc	✗	Twitter	Classical machine learning algorithms	89% Accuracy
^33^	Examine effect of depression on people’s Twitter language	✗	Twitter	Transformer-based classifiers	78.9% Accuracy
^34^	Predicting mental health status of Reddit users	✗	SMHD Dataset from Reddit by^41^	Hierarchical attention network	HAN performed better in classification of Depression, ADHD, Anxiety, Biolar when compared with other classifiers as compared to PTSD, Autism, OCD, Schizo, Eating
^42^	Identification of PTSD among cancer survivors	✗	Twitter	CNN	91.29% Accuracy
^35^	Screening of Twitter users for depression and PTSD	✗	Twitter	Lexical decision lists of n-grams	Average precision in the range of 0.70–0.76
^36^	Identification of depression with Temporal Measures of Emotions	✗	Twitter	Emotion and temporal features with RF	91.81% Accuracy
^43^	Classification of mental illness	✗	Reddit	Traditional machine learning, deep learning and transfer learning	83% Accuracy with RoBERTa
^12^	Prediction of mental illness	✗	Twitter	Classical machine learning algorithms	0.89 AUC with RF
^44^	Identification of PTSD among military personnel	✗	Self-reported service exposures and a range of validated self-report measures	Classical machine learning algorithms	97% Accuracy with RF
^45^	Prediction of PTSD survivors Northern Ugandan rebel war	✗	Counsellors visited residents of the former IDP camps and communities at their homes	RF-CI and LASSO	77.25% Accuracy with RF-CI
^46^	Classifying PTSD in US veterans	✗	Speech samples from warzone-exposed veterans	RF	0.954 AUC
^47^	Assessment of PTSD in military personnel	✗	Audio recordings of interviews	GB, DT, NN and boosting	77% Accuracy
This work	Identification of PTSD among Covid survivors	$\checkmark$	Twitter	NB, kNN, SVM, RF	83.29% Accuracy with SVM

In another study by^43^, the classification of mental illness from social media texts using deep learning and transfer learning was investigated. The authors aimed to develop a machine learning model to identify the presence of mental illness in text data from social media platforms. The model was trained on a dataset of social media texts annotated for mental illness and evaluated using multiple metrics. The results showed that the transfer learning approach outperformed traditional deep learning methods in terms of accuracy in classifying mental illness in social media texts. This study highlights the potential of deep learning and transfer learning for mental health screening and intervention through social media platforms.

A number of studies have been conducted using machine learning algorithms to predict PTSD and depression in various populations. For example, Reece et al. used RF algorithm to analyze 243,000 Twitter posts related to PTSD and achieved an AUC score of 0.89 in predicting the disorder^12^.

Another study conducted by Leightley et al. focused on identifying PTSD among military personnel in the UK by applying machine learning techniques. They achieved an accuracy of 97% with RF^44^. Papini et al. used gradient-boosted decision trees to predict PTSD in 110 patients with the disorder and 231 trauma-exposed controls, achieving an accuracy of 78%^48^. Similarly, Conrad et al. applied RF using Conditional Interference (RF-CI) and Least Absolute Shrinkage and Selection Operator (LASSO) to predict PTSD survivors of a civil war in Uganda, with RF achieving the highest accuracy of 77.25%^45^.

Marmar et al. used RF to predict PTSD with an accuracy of 89.1% with an AUC of 0.954 from audio recordings of warzone-exposed veterans^46^. Vergyri et al. used Gaussian backend (GB), decision trees (DT), neural network (NN) classifiers, and boosting to predict PTSD from audio recordings of war veterans, obtaining an overall accuracy of 77%^47^.

According to^42^, a noteworthy investigation was conducted to detect PTSD among cancer survivors using Twitter data. The researchers utilized a convolutional neural network (CNN) to learn the representations of the input tweets containing the keywords “cancer” and “PTSD” to identify cancer survivors with PTSD. The results demonstrated that the proposed CNN was effective in detecting PTSD among cancer survivors and outperformed the baselines. The authors suggested that it is crucial to evaluate and treat PTSD in cancer survivorship care, and social media can act as an early warning system for PTSD in cancer survivors. The study emphasizes the importance of early detection and treatment of PTSD in cancer survivors.

Our research builds on earlier studies by focusing on PTSD in people who have survived COVID-19, aiming to better understand the psychological effects of the pandemic. To address the mental health needs that haven’t been met for these survivors, we have used a unique approach. As shown in Table 1, we compare our study to previous work to highlight how our investigation is different. Unlike other research that looks at various groups, we specifically analyze how COVID-19 has impacted mental health using information gathered from Twitter. Our method successfully pinpointed PTSD in 83.29% of cases, proving to be a valuable tool in understanding how this global crisis affects mental well-being.

## Methodology

**Figure 1:**
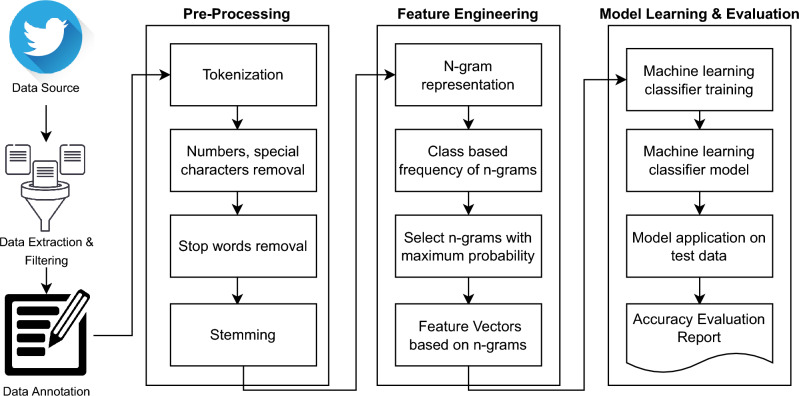
Proposed methodology.

After thoroughly reviewing state-of-the-art techniques, we have proposed a classification framework as shown in Fig. 1.

The stages of our proposed system are as follows: (i) data extraction and filtering (ii) data annotation based on ICD-11 guidelines (iii) preprocessing and splitting data into train and test dataset (iv) extraction of features and (v) training and evaluation of our ML model.

### Data extraction

The first step of data extraction is to identify those users who, mentioned on twitter that they were *covid positive*. We collected the data including tweets from Twitter using official Twitter API for academic research with the search query “#Covidpositive OR #Covidsurvivor OR #CovidFree OR #CovidRecovered OR #ConqueredCovid OR #DefeatedCovid OR #OvercameCovid”. The data was collected from 01-March-2020 till 30-November-2021. We ended up with 90,330 usernames who posted tweets using either of these hashtags during this period. However, the unique usernames were 70,646.

**Figure 2:**
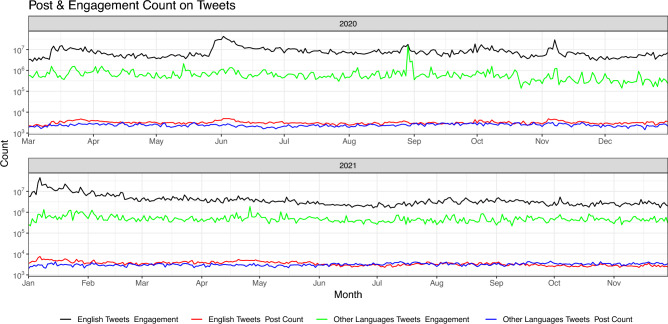
Tweets and engagement count of english and other languages.

We applied the sample size (*n*) calculation formula provided by^49^ given below as (1), on population (*N*) of 70,646.

1 $$\begin{aligned} n = \frac{N}{1 + N(e)^{2}} \end{aligned}$$

where *e* is the margin of error and we choose it to be 5%. By applying Eq. 1, we got $\approx$ 177 users. We randomly choose 177 users from the previously extracted data, and we extracted the tweets timeline of these users using the aforementioned timeline. We were able to extract 3,958,836 tweets ($\approx$ 3.96 Million) out of which 2,155,577 Tweets were in English language ($\approx$ 2.15 Million). Furthermore, the focus of the model was solely on the text content of the tweets for classification purposes, without relying on any demographic information. This data is visualized in Fig. 2 for both, years and categories.

**Table 2:** Set of keywords to filter the tweets.

Keyword	Count	Keyword	Count	Keyword	Count
Re-experiencing					
Flashbacks	36	Intrusions	6	Panic	2102
Nightmares	155	Preoccupied	23	Vivid dreams	3
Hyperarousal					
Agitated	76	Hypervigilant	1	Irritable	5
Startle	30				
Avoidance					
Avoid	3,381	Avoidance	38		
Other affective and biological symptoms related to PTSD					
Anxiety	934	Appetite	93	Emotion	2557
Depressed	265	Insomnia	77	Fatigue	314
Low	72,942	Negative thoughts	12	Sleep	4218
Suicidal thoughts	27	Trauma	989	Weight	1363

To filter the data, we used a set of keywords inline with the ICD-11 guidelines. The breakdown of tweets against each keyword is mentioned in the Table 2.

**Figure 3:**
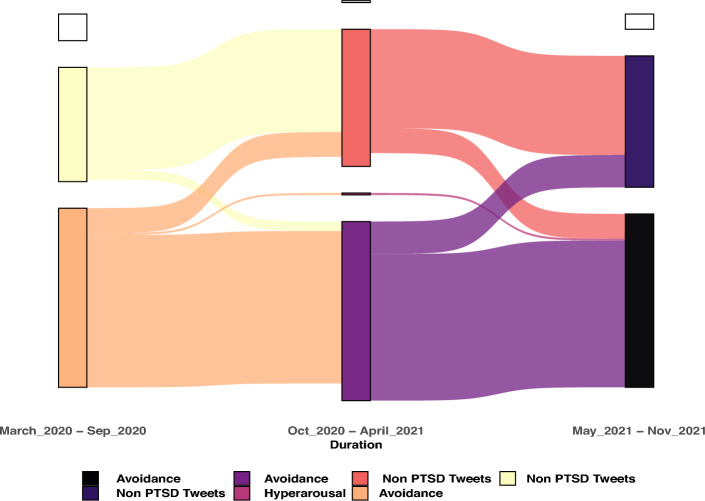
Flow of users across PTSD categories over time.

To further understand the posting behavior of users about previously mentioned PTSD categories, we have visualized the flow of users, in Fig. 3, across three intervals of seven months each from our selected timeline.

The intervals include data from (i) March 2020–September 2020 (ii) October 2020–April 2021 (iii) May 2021–November 2021. We see a large fraction of users contributing to *Avoidance* category, and then the second most contribution is to *Non PTSD Tweets*, i.e., which did not belong to any of the previously mentioned categories. In fact, these two remains the only categories about which most users have posted and they have further continued posting about either of them. The only small presence is of *Hyperarousal* in second interval. The interesting fact to be noted is that a large fraction of users have remained in their respective categories across all intervals, however, a small number of users switched their categories in second interval and approximately the same number of users came back in their initial category. We can say that majority of the users were found to not have PTSD symptoms after they were diagnosed with Covid.

**Figure 4:**
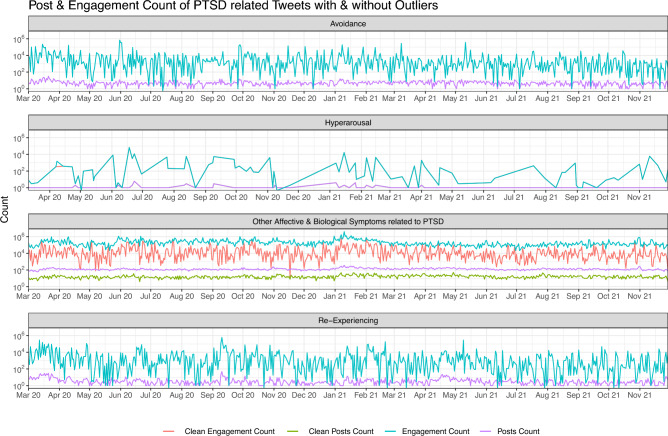
Category wise tweets and engagement breakdown.

The total of tweets after filtering was 89,647 as per the breakdown mentioned in Table 2. To make sure that we do not have too much and too low representation of one particular keywords, we chose to calculate the 5th and 96th percentile of these numbers to remove the too low and too high, respectively, occurrence of tweets against keywords. The 5th percentile is 3.3 for this data, and 96th percentile is 9715.92. Based on these values, we will exclude values lower than 5th percentile, i.e, *Hypervigilant* greater than 96th percentile, i.e, *Low*, therefore, the final set of tweets were 16,704, used for the annotation of data. This breakdown of data for categories mentioned in Table 2 and after removing the outliers in aforementioned categories, is shown in Fig. 4. Since the change is supposed to occur in only two categories where these values belong, that’s why only two of them contain *Clean Engagement Count* and *Clean Posts Count*.

### Ethical considerations

We recognize the importance of maintaining privacy and sensitivity when working with mental health data, particularly when utilizing user-generated content such as tweets. To address these concerns, all tweets used in our study were anonymized to ensure the protection of user identities. No personally identifiable information was retained in our analysis and hence we cannot trace back any user after our analysis.

### Data pre-processing

Once the data had been filtered, we proceeded to pre-process it in order to eliminate any extraneous noise. To this end, we undertook the following steps:

1. We began by selecting a Covid-PTSD category from the training data.
2. Next, we broke down the tweets within this category into tokens using delimiters.
3. After tokenizing the tweets, we sanitized them to remove any non-letter characters, such as punctuation marks, quotes, numbers, and special characters, among others.
4. We then eliminated all stop words, which are typically less informative, from the dataset. To do this, we utilized NLTK-based stop word lists and also generated our own stop word lists.
5. After implementing step four, we utilized a Porter stemmer to perform text stemming. This step is vital in reduce or minimize the dimensions of the features since a word can exist in multiple forms with different meanings in natural language (e.g., singular and plural). By stemming the words, we reduced them to their base form.
6. Steps 1-5 were repeated for both classes.

### Classification algorithms

In this article classical machine learning algorithms are used for classification problems. All the four algorithms are discussed in this section.

- **Support Vector Machine (SVM)** is a commonly used technique for text categorization^50,51^. It employs multidimensional hyperplanes to accurately differentiate between different labels or classes^52^. SVMs are particularly useful in high-dimensional spaces, making them the most practical classifier for such scenarios. Additionally, SVMs offer fair predictive performance even with small datasets due to their relative simplicity and versatility in handling a wide range of classification problems. SVMs are widely used in brain disorder research utilizing multivoxel pattern analysis (MVPA) due to their simplicity and lower risk of overfitting. In recent times, SVMs have been applied in precision psychiatry, particularly in the diagnosis and prognosis of brain diseases like Alzheimer’s, schizophrenia, and depression^53^.
- **Naïve Bayes (NB)** is a machine learning algorithm that utilizes probability to classify data. It calculates the likelihood of a given piece of text belonging to a particular class based on the computed class labels. The classifier has been successfully employed in several studies for text classification^54–57^. We chose this classifier for its ease of use and superior performance in earlier studies^58,59^. The algorithm performs a sequence of probabilistic computations to determine the best-fitted classification for a given piece of data. Suppose *x* is a set of *n* attributes, such that $X = {x_{1}, x_{2}, x_{3},...,x_{n}}$ where *X* represents the evidence, and *H* represents the hypothesis that the data sample *X* belongs to a certain class *C*. The likelihood that the hypothesis *H* holds given the evidence *X* can be computed using Equation 2. The Bayes theorem explains the preceding logic as follows:
  2 $$\begin{aligned} P(H|X) = (P(P|H) P(H)) / P(X) \end{aligned}$$
- **K-Nearest Neighbors (KNN)** is an instance-based machine learning classifier based on the concept of similarity. It determines a class’s similarity to a feature using the Euclidean equation and the value of *K*. The algorithm stores all cases and uses a similarity score to identify new examples. The similarity between a new text and the training data is recognized and calculated, and the texts with the highest similarity are chosen. Finally, the class is identified using *K* neighbors. However, when *K* is a large value, the computation required to determine the most suitable class becomes difficult^60,61^.
- **Random Forest (RF)** is a supervised learning approach which was proposed by Ho in 1995^62^. It involves constructing multiple decision trees that work in unison, with decision trees serving as the building blocks. During pre-processing, nodes are selected for the decision trees. A random subset of features is used to determine the best feature, and a decision tree is created based on the input vector to classify new objects. Every decision tree is used for classification, and the algorithm assigns tree votes to each class. The class with the most votes from all the decision trees in the forest is selected as the final classification. RF has many advantages over other classifiers such as SVM and NB. For example, RF can handle noisy and missing data, it is robust to overfitting and can work well with high-dimensional data^58^. RF can also provide information about the relative importance of the features used in classification, which is useful in feature selection and understanding the underlying data structure. In the field of text classification such as sentiment analysis, categorization of news posts, spam filtering etc., RF has been widely used with significant results^58,63^. Additionally, RF has been applied in feature selection for text classification by using the Gini impurity index, which measures the importance of a feature by the reduction in the impurity of the resulting classification tree^62^.

## Experimental evaluation

We performed our experiments on a specific dataset collected through the process mentioned in section “Data extraction”. Here in this section we discuss the details about annotating that data and the evaluation metrics we used to evaluate the results of our proposed model.

### Data annotation and metrics of evaluation

We used ICD-11 criteria for diagnosing PTSD. Being infected with COVID-19 was identified as a triggering event, and then we looked for symptoms under three core domains outlined in ICD-11^28^ including re-experiencing, hyperarousal, and avoidance behavior. Apart from these three core domains, we also looked at other affective or mood symptoms, its impact, and the treatment availed by the population being studied. Once tweet timelines were extracted once they were identified as *“Covid Positive”* according to the criteria mentioned in section “Data extraction” PTSD keywords mentioned in Table 2 were used further to filter the most relevant tweets according to the ICD-11 criteria.

- Tweets which had both their COVID-19 status as well as one of the PTSD keywords mentioned were considered as “PTSD Positive”.
- All those tweets that mentioned PTSD keywords but in relation to any other event rather than COVID-19 were not taken into consideration and were deemed “PTSD Negative”.

In the Table 3, we provide a sample of Tweets for both of the classes.

**Table 3:** Classes with example Tweets.

Label	Message
PTSD positive	“Yes, covid has affected my mental health - Trump had a worse affect. I was already seriously anxious; depressed because of Trump; then his lack of action on covid made it so much worse.”
	“The tragedy, trauma, and death from COVID is wiping out generations of families. It is just perverse.”
	“Listening to friends telling horror stories during the second wave of covid in Delhi. 2 of them lost their mothers. One died due to unprofessional greedy attitude of the doc. She wept telling how her mom with comorbidities suffered. I’m filled with emotions I can’t explain.”
PTSD negative	“A new day, When anxiety is replaced by a sense of relief”.
	“Good people still exist! I lost my wallet yesterday and went into full panic mode, canceling everything. I just got a call from the person who found it. Cash, credit cards, license—everything is still there.”
	“People who regularly help others are significantly happier and less likely to become depressed as they get older.”

Based on these guidelines, we annotated the dataset of 16,704 tweets and only 1,092 of them found to be *PTSD Positive*. To keep the dataset balanced, from rest of tweets we took only 1,092 *PTSD Negative* tweets and used it for our classifiaction model. To train and test our proposed model, we kept 80% of data to train and 20% for test.

Additionally, we computed several performance metrics to assess the efficacy of our proposed approach. These metrics include accuracy, precision, recall, and F1-score, which are frequently used in information retrieval to evaluate the effectiveness of models. As our study involves binary-label classification, we followed the metrics proposed by^64^. This step was necessary to validate our findings.

Given a set $C = \{ c_1, c_2 \}$ of *class labels*, for each class $c_j$ we define the following counts:

In a binary classification problem, evaluating the performance of the classifier against each class $c_j$ is important. For this purpose, we can use four metrics, which are commonly used in evaluation of classifiers for binary classification problems. These metrics are as follows:

- True Positives ($TP_j$): predicted values that are accurately classified as positive.
- False Positives ($FP_j$): predicted values that are wrongly classified as positive.
- False Negatives ($FN_j$): predicted values that are wrongly classified as negative.
- True Negatives ($TN_j$): predicted values that are accurately classified as negative.

These metrics provide a quantitative measure of how well the classifier is performing for each class, and can be used to identify areas for improvement in the classification model.

- $Accuracy_j$: is defined as the proportion of properly predicted observations with class $c_j$ to total number of observations. The mathematical formula is as follows:
  3 $$\begin{aligned} Accuracy_j = \frac{TP_j + TN_j}{TP_j + TN_j + FP_j + FN_j}. \end{aligned}$$
- $Precision_j$: is defined as the ratio of correctly predicted observations with class $c_j$ to the total number of correctly predicted observations. The mathematical formula is as follows:
  4 $$\begin{aligned} Precision_j = \frac{TP_j}{TP_j + FP_j} \end{aligned}$$
- $Recall_j$: is defined as the proportion of correctly predicted observations labeled with class $c_j$ to the total number of observations in a class. The mathematical formula is as follows:
  5 $$\begin{aligned} Recall_j = \frac{TP_j}{TP_j + FN_j} \end{aligned}$$
- $F1-measure_j$: is the harmonic average of $Recall_j$ and $Precision_j$. The mathematical formula is as follows:
  6 $$\begin{aligned} F1\text {-}measure_j = \frac{Precision_j \times Recall_j}{Precision_j + Recall_j} \times 2. \end{aligned}$$

While performing classification in the case of binary classes, we take the average of all the numbers calculated for each class.

Consequently,

7 $$\begin{aligned}{} & {} Accuracy = \frac{(Accuracy_1 + Accuracy_2)}{2} \end{aligned}$$

8 $$\begin{aligned}{} & {} Precision = \frac{(Precision_1 + Precision_2)}{2} \end{aligned}$$

9 $$\begin{aligned}{} & {} Recall = \frac{(Recall_1 + Recall_2)}{2} \end{aligned}$$

10 $$\begin{aligned}{} & {} F1\text {-}measure = \frac{(F1\text {-}measure_1 + F1\text {-}measure_2)}{2} \end{aligned}$$

In addition to these metrics, the Area Under the Curve (AUC) is commonly used as a performance metric to evaluate the classifier’s ability to distinguish between positive and negative classes. AUC represents the area under the Receiver Operating Characteristic (ROC) curve, which plots the true positive rate (TPR) against the false positive rate (FPR) at various threshold settings.

The equation for AUC calculation can be written as:

11 $$\begin{aligned} \text {AUC} = \frac{1}{2} \sum _{i=1}^{N} \left( \text {TPR}_i + \text {TPR}_{i-1} \right) \times \left( \text {FPR}_i - \text {FPR}_{i-1} \right) \end{aligned}$$

where $\text {TPR}_i$ is the True Positive Rate at the *i*th threshold, $\text {FPR}_i$ is the False Positive Rate at the *i*th threshold, and *N* is the number of thresholds.

In the next section, results are reported and discussed by executing the proposed framework on our dataset.

## Experiments and comparison of classifiers

Let us consider the following feature patterns mentioned in Table 4 against which we have computed our results using previously mentioned classifiers.

**Table 4:** Feature patterns with their abbreviations.

Abbreviation	Feature pattern
**U**	Unigrams
**B**	Bigrams
**T**	Trigrams
**Q**	Quadgrams
**U** $\times$ **B**	The product of two sets U and B using the Cartesian method
**B** $\times$ **T**	The product of two sets B and T using the Cartesian method
**T** $\times$ **Q**	The product of two sets T and Q using the Cartesian method
**U** $\times$ **B** $\times$ **T**	The product of three sets U, B and T using the Cartesian method
**U** $\times$ **B** $\times$ **T** $\times$ **Q**	The product of four sets U, B, T and using the Cartesian method

Using the above feature patterns in combination with aforementioned classifiers in section “Classification algorithms”, we performed our experimentation and evaluated the results based on evaluation metrics mentioned in section “Data annotation and metrics of evaluation”. In Table 5, we reported our findings using NB classifier.

**Table 5:** Results obtained by NB.

Pattern	Accuracy	Precision	Recall	F1-measure	AUC
**U**	81.38	81.94	81.38	81.15	81.06
**B**	61.58	62.68	61.58	59.63	60.78
**T**	56.56	69.27	56.56	45.17	54.80
**Q**	55.13	72.72	55.13	41.37	53.25
**U** $\times$ **B**	81.62	82.06	81.62	81.42	81.33
**B** $\times$ **T**	61.34	62.35	61.34	59.42	60.56
**T** $\times$ **Q**	56.56	69.27	56.56	45.17	54.80
**U** $\times$ **B** $\times$ **T**	**81.86**	82.27	81.86	81.67	**81.58**
**U** $\times$ **B** $\times$ **T** $\times$ **Q**	81.62	82.06	81.62	81.42	81.33

Significant values are given in bold.

**Table 6:** Results obtained by kNN.

**Pattern**	Accuracy	Precision	Recall	F1-measure	AUC
**U**	**76.61**	76.60	76.61	76.56	**76.55**
**B**	66.11	66.43	66.11	66.10	66.27
**T**	47.97	23.01	47.97	32.42	50.00
**Q**	47.97	23.01	47.97	32.42	50.00
**U** $\times$**B**	75.18	75.17	75.18	75.13	75.12
**B** $\times$**T**	55.61	60.58	55.61	46.23	54.00
**T** $\times$**Q**	47.97	23.01	47.97	32.42	50.00
**U** $\times$**B** $\times$**T**	74.22	74.33	74.22	74.22	74.28
**U** $\times$**B** $\times$**T** $\times$**Q**	75.42	75.58	75.42	75.42	75.50

Significant values are given in bold.

NB achieved the maximum accuracy of 81.86% with **UxBxT** as feature pattern. Meanwhile, it is notable that accuracy is > 81% in all combinations where **U** is present. Otherwise, the performance have declined.

In Table 6, the results computed using kNN are mentioned.

Similar to NB, kNN achieved highest accuracies with **U** or its combination with other feature patterns, achieving accuracy > 74% in its combinations. The maximum was 76.61% with **U**.

Tables 7 and 8 report the findings by SVM and RF, respectively.

**Table 7:** Results obtained by SVM.

Pattern	Accuracy	Precision	Recall	F1-measure	AUC
**U**	**83.29**	83.44	83.29	83.29	**83.38**
**B**	61.58	63.93	61.58	58.50	60.57
**T**	50.12	57.58	50.12	40.01	51.87
**Q**	48.21	57.75	48.21	33.32	50.21
**U** $\times$ **B**	82.10	82.10	82.10	82.05	82.02
**B** $\times$ **T**	61.58	68.33	61.58	55.93	60.22
**T** $\times$ **Q**	49.40	54.20	49.40	39.57	51.12
**U** $\times$ **B** $\times$ **T**	81.86	81.88	81.86	81.80	81.75
**U** $\times$ **B** $\times$ **T** $\times$ **Q**	81.86	81.90	81.86	81.79	81.73

Significant values are given in bold.

**Table 8:** Results obtained by RF.

Pattern	Accuracy	Precision	Recall	F1-measure	AUC
**U**	78.28	78.54	78.28	78.28	78.41
**B**	61.10	65.61	61.10	56.36	59.86
**T**	48.69	75.21	48.69	33.93	50.69
**Q**	48.21	75.10	48.21	32.93	50.23
**U** $\times$ **B**	**80.67**	81.13	80.67	80.66	**80.86**
**B** $\times$ **T**	60.62	66.99	60.62	54.65	59.25
**T** $\times$ **Q**	48.69	75.21	48.69	33.93	50.69
**U** $\times$ **B** $\times$ **T**	80.43	81.08	80.43	80.42	80.67
**U** $\times$ **B** $\times$ **T** $\times$ **Q**	79.95	80.89	79.95	79.92	80.25

Significant values are given in bold.

Among all the classifiers we have used in this study, SVM has outperformed other three with highest classification accuracy of 83.29%. Meanwhile, NB comes second with 81.86%, whereas RF and kNN are at third and fourth place with 80.67% and 76.61% accuracy respectively. The findings by SVM and NB are consistent with those by RF and kNN in terms of better accuracy with feature patterns **U** or its cartesian product with another pattern. All classifiers have performed better with **U** or **UxB** or **UxBxT** or **UxBxTxQ**. When **U** is not among the feature patterns, the accuracy has declined among all classifiers. While it is evident that our preferred classification model had 83.29% accuracy, it is important to note where the model misclassified the tweets. As mentioned before in the guidelines that we followed for annotation, states that the tweets which have both, i.e., PTSD related keywords and information related to Covid-19 were labelled as PTSD Positive and others as PTSD Negative. And we encountered instances where tweets containing keywords related to PTSD were labeled as PTSD Negative if they were not specifically related to Covid-19. This approach occasionally resulted in misclassifications, particularly false positives where tweets were incorrectly identified as PTSD positive.

**Figure 5:**
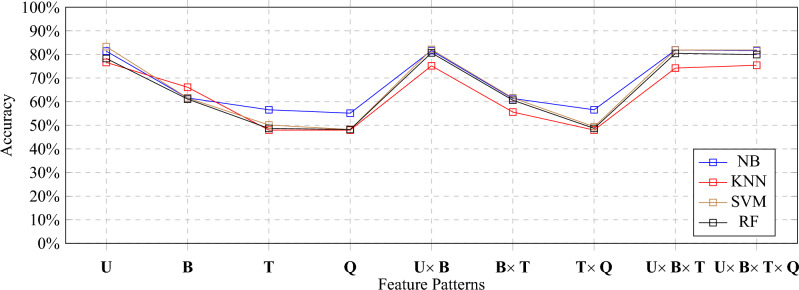
Accuracy comparison of all classifiers.

Findings reported in Tables 5, 6, 7 and 8 are visualized in Fig. 5 for better comparison of results.

The best performing algorithm i.e., SVM, which got highest accuracy of i.e., 83.29% with **U**, it turns out that it did not perform so well with **B** and **BxT** where the accuracy declined significantly. Similarly, as reported in^58,59^, *U* gives us low computational cost because of less number of features produced as a result of applying TF-IDF, it is a preferred choice for the model to be used for final act of classification.

## Conclusion

In this study, we performed our analysis to understand the post COVID-19 mental health dynamics and tweets consumption of COVID-19 positive users. We identified them by using a set of hashtags reflecting the positive diagnosis of Covid. We then extracted their Twitter timelines and performed our analysis on more than 3.96 Million pieces of content produced between March 2020 and November 2021. Our findings suggest that post circulation related to “Other Affective & Biological Symptoms related to PTSD” category is higher than other categories. However, we noticed that a large fraction of users shifted their behavior from “Avoidance” to “Non PTSD Related” and vice versa. We used ICD-11 guidlines to filter and annotate our tweets and developed a machine learning based classification model to segregate our tweets into either PTSD positive or PTSD negative. We got our best results with SVM on unigram as feature pattern with 83.29% accuracy. We also acknowledge that our study’s concentration on English-language tweets may restrict the usefulness of our model for other languages or platforms with different ways of expression. We’re taking this into account for future research plans. In future, we further aim to extend this work by (i) extending the dataset of PTSD Positve tweets and (ii) extracting all the replies/comments on them to (iii) create a model to effectively understand/classify the sentiments of users on those posts.

## Data Availability

The datasets generated during and/or analysed during the current study are available from the corresponding author on reasonable request.

## References

[1] WHO. WHO Coronavirus (COVID-19) Dashboard. https://covid19.who.int/ (2022). [Online; accessed 13-March-2023].

[2] UNESCO. Education: from disruption to recovery. https://en.unesco.org/covid19/educationresponse (2022). [Online; accessed 15-January-2022].

[3] Inskip, H., Harris, C. & Barraclough, B. Lifetime risk of suicide for affective disorder, alcoholism and schizophrenia. *Br. J. Psychiatry* **172**, 35–37 (1998). 9534829 10.1192/bjp.172.1.35

[4] San Too, L. *et al.* The association between mental disorders and suicide: a systematic review and meta-analysis of record linkage studies. *J. Affect. Disord.* textbf259, 302–313 (2019). 10.1016/j.jad.2019.08.054 31450139

[5] Yoshikawa, E., Taniguchi, T., Nakamura-Taira, N., Ishiguro, S. & Matsumura, H. Factors associated with unwillingness to seek professional help for depression: A web-based survey. *BMC. Res. Notes* **10**, 1–6 (2017). 29202791 10.1186/s13104-017-3010-1 PMC5716254

[6] Baker, S. R., Bloom, N., Davis, S. J. & Terry, S. J. *Covid-induced economic uncertainty* (Tech. Rep, National Bureau of Economic Research, 2020).

[7] Nicola, M. *et al.* The socio-economic implications of the coronavirus pandemic (covid-19): A review. *Int. J. Surg.* **78**, 185–193 (2020). 32305533 10.1016/j.ijsu.2020.04.018 PMC7162753

[8] Xiong, J. *et al.* Impact of covid-19 pandemic on mental health in the general population: A systematic review. *J. Affect. Disord.* **277**, 55–64 (2020). 32799105 10.1016/j.jad.2020.08.001 PMC7413844

[9] Brooks, S. K. *et al.* The psychological impact of quarantine and how to reduce it: Rapid review of the evidence. *The lancet* **395**, 912–920 (2020). 10.1016/S0140-6736(20)30460-8 PMC7158942 32112714

[10] Czeisler, M. É. *et al.* Mental health, substance use, and suicidal ideation during a prolonged covid-19-related lockdown in a region with low sars-cov-2 prevalence. *J. Psychiatr. Res.* **140**, 533–544 (2021). 34174556 10.1016/j.jpsychires.2021.05.080 PMC8177437

[11] Rehman, I. U. *et al.* Features of mobile apps for people with autism in a post covid-19 scenario: Current status and recommendations for apps using ai. *Diagnostics* **11**, 1923 (2021). 34679621 10.3390/diagnostics11101923 PMC8535154

[12] Reece, A. G. *et al.* Forecasting the onset and course of mental illness with twitter data. *Sci. Rep.* **7**, 1–11 (2017). 29021528 10.1038/s41598-017-12961-9 PMC5636873

[13] Paparrizos, J., White, R. W. & Horvitz, E. Screening for pancreatic adenocarcinoma using signals from web search logs: Feasibility study and results. *J. Oncol. Pract.* **12**, 737–744 (2016). 27271506 10.1200/JOP.2015.010504

[14] Christakis, N. A. & Fowler, J. H. Social network sensors for early detection of contagious outbreaks. *PLoS ONE* **5**, e12948 (2010). 20856792 10.1371/journal.pone.0012948 PMC2939797

[15] Schmidt, C. W. Trending now: using social media to predict and track disease outbreaks (2012). 10.1289/ehp.120-a30 PMC3261963 22214548

[16] Moreno, M. A., Christakis, D. A., Egan, K. G., Brockman, L. N. & Becker, T. Associations between displayed alcohol references on facebook and problem drinking among college students. *Arch. Pediatr. Adolesc. Med.* **166**, 157–163 (2012). 21969360 10.1001/archpediatrics.2011.180 PMC3266463

[17] De Choudhury, M., Kiciman, E., Dredze, M., Coppersmith, G. & Kumar, M. Discovering shifts to suicidal ideation from mental health content in social media. In *Proceedings of the 2016 CHI conference on human factors in computing systems*, 2098–2110 (2016). 10.1145/2858036.2858207 PMC5659860 29082385

[18] De Choudhury, M., Gamon, M., Counts, S. & Horvitz, E. Predicting depression via social media. In *Seventh international AAAI conference on weblogs and social media* (2013).

[19] Katikalapudi, R., Chellappan, S., Montgomery, F., Wunsch, D. & Lutzen, K. Associating internet usage with depressive behavior among college students. *IEEE Technol. Soc. Mag.* **31**, 73–80 (2012). 10.1109/MTS.2012.2225462

[20] Moreno, M. A. *et al.* Feeling bad on facebook: Depression disclosures by college students on a social networking site. *Depress. Anxiety* **28**, 447–455 (2011). 21400639 10.1002/da.20805 PMC3110617

[21] Coppersmith, G., Harman, C. & Dredze, M. Measuring post traumatic stress disorder in twitter. In *Eighth international AAAI conference on weblogs and social media* (2014).

[22] Park, M., Cha, C. & Cha, M. Depressive moods of users portrayed in twitter. In *Proceedings of the 18th ACM International Conference on Knowledge Discovery and Data Mining, SIGKDD 2012*, 1–8 (2012).

[23] Reece, A. G. & Danforth, C. M. Instagram photos reveal predictive markers of depression. *EPJ Data Sci.* **6**, 15 (2017). 10.1140/epjds/s13688-017-0110-z

[24] Ferrari, A. *et al.* Global variation in the prevalence and incidence of major depressive disorder: A systematic review of the epidemiological literature. *Psychol. Med.* **43**, 471–481 (2013). 22831756 10.1017/S0033291712001511

[25] Gwynn, R. C. *et al.* Prevalence, diagnosis, and treatment of depression and generalized anxiety disorder in a diverse urban community. *Psychiatr. Serv.* **59**, 641–647 (2008). 18511584 10.1176/ps.2008.59.6.641

[26] Campbell, D. G. *et al.* Prevalence of depression-ptsd comorbidity: Implications for clinical practice guidelines and primary care-based interventions. *J. Gen. Intern. Med.* **22**, 711–718 (2007). 17503104 10.1007/s11606-006-0101-4 PMC2219856

[27] Munro, C. G., Freeman, C. P. & Law, R. General practitioners’ knowledge of post-traumatic stress disorder: A controlled study. *Br. J. Gen. Pract.* **54**, 843–847 (2004). 15527610 PMC1324918

[28] WHO. International Statistical Classification of Diseases and Related Health Problems (ICD). https://www.who.int/standards/classifications/classification-of-diseases (2022). [Online; accessed 28-January-2022].

[29] American Psychiatric Association, D., Association, A. P. *et al.**Diagnostic and statistical manual of mental disorders: DSM-5*, vol. 5 (American psychiatric association Washington, DC, 2013).

[30] Van der Kolk, B. A., Pelcovitz, D., Roth, S., Mandel, F. S. *et al.* Dissociation, somatization, and affect dysregulation: The complexity of adaption to trauma. *Am. J. Psychiatry* (1996). 10.1176/ajp.153.7.83 8659645

[31] Wang, Y.-X. *et al.* Factors associated with post-traumatic stress disorder of nurses exposed to corona virus disease 2019 in china. *Medicine* **99** (2020). 10.1097/MD.0000000000020965 PMC7328992 32590808

[32] Joshi, D. J., Makhija, M., Nabar, Y., Nehete, N. & Patwardhan, M. S. Mental health analysis using deep learning for feature extraction. In *Proceedings of the ACM India Joint International Conference on Data Science and Management of Data*, pp. 356–359 (2018).

[33] Zhang, Y. *et al.* Monitoring depression trends on twitter during the covid-19 pandemic: Observational study. *JMIR Infodemiol.* **1**, e26769 (2021). 10.2196/26769 PMC8330892 34458682

[34] Sekulić, I. & Strube, M. Adapting deep learning methods for mental health prediction on social media. arXiv preprint arXiv:2003.07634 (2020).

[35] Pedersen, T. Screening twitter users for depression and ptsd with lexical decision lists. In *Proceedings of the 2nd workshop on computational linguistics and clinical psychology: From linguistic signal to clinical reality*, 46–53 (2015).

[36] Chen, X., Sykora, M. D., Jackson, T. W. & Elayan, S. What about mood swings: Identifying depression on twitter with temporal measures of emotions. *In Companion Proceedings of the The Web Conference* **2018**, 1653–1660 (2018).

[37] Coppersmith, G., Dredze, M. & Harman, C. Quantifying mental health signals in twitter. In *Proceedings of the workshop on computational linguistics and clinical psychology: From linguistic signal to clinical reality*, pp. 51–60 (2014).

[38] Coppersmith, G., Dredze, M., Harman, C. & Hollingshead, K. From adhd to sad: Analyzing the language of mental health on twitter through self-reported diagnoses. In *Proceedings of the 2nd workshop on computational linguistics and clinical psychology: From linguistic signal to clinical reality*, pp. 1–10 (2015).

[39] Coppersmith, G., Dredze, M., Harman, C., Hollingshead, K. & Mitchell, M. Clpsych 2015 shared task: Depression and ptsd on twitter. In *Proceedings of the 2nd workshop on computational linguistics and clinical psychology: From linguistic signal to clinical reality*, 31–39 (2015).

[40] Weerasinghe, J., Morales, K. & Greenstadt, R. Linguistic indicators of mental health status on twitter“ because... i was told... so much”. *Proc. Priv. Enhancing Technol.* **2019**, 152–171 (2019).

[41] Cohan, A. *et al.* Smhd: A large-scale resource for exploring online language usage for multiple mental health conditions. arXiv preprint arXiv:1806.05258 (2018).

[42] Ismail, N. H., Liu, N., Du, M., He, Z. & Hu, X. A deep learning approach for identifying cancer survivors living with post-traumatic stress disorder on twitter. *BMC Med. Inform. Decis. Mak.* **20**, 1–11 (2020). 33317508 10.1186/s12911-020-01272-1 PMC7734710

[43] Ameer, I., Arif, M., Sidorov, G., Gòmez-Adorno, H. & Gelbukh, A. Mental illness classification on social media texts using deep learning and transfer learning. arXiv preprint arXiv:2207.01012 (2022).

[44] Leightley, D., Williamson, V., Darby, J. & Fear, N. T. Identifying probable post-traumatic stress disorder: Applying supervised machine learning to data from a uk military cohort. *J. Ment. Health* **28**, 34–41 (2019). 30445899 10.1080/09638237.2018.1521946

[45] Conrad, D. *et al.* Does trauma event type matter in the assessment of traumatic load?. *Eur. J. Psychotraumatol.* **8**, 1344079 (2017). 28804594 10.1080/20008198.2017.1344079 PMC5533143

[46] Marmar, C. R. *et al.* Speech-based markers for posttraumatic stress disorder in us veterans. *Depress. Anxiety* **36**, 607–616 (2019). 31006959 10.1002/da.22890 PMC6602854

[47] Vergyri, D. *et al.* Speech-based assessment of ptsd in a military population using diverse feature classes. In *Sixteenth annual conference of the international speech communication association* (Citeseer, 2015).

[48] Papini, S. *et al.* Ensemble machine learning prediction of posttraumatic stress disorder screening status after emergency room hospitalization. *J. Anxiety Disord.* **60**, 35–42 (2018). 30419537 10.1016/j.janxdis.2018.10.004 PMC6777842

[49] Taherdoost, H. Determining sample size; how to calculate survey sample size. *Int. J. Econ. Manag. Syst.* **2** (2017).

[50] Gelbard, R., Ramon-Gonen, R., Carmeli, A., Bittmann, R. M. & Talyansky, R. Sentiment analysis in organizational work: Towards an ontology of people analytics. *Expert. Syst.* **35**, e12289 (2018). 10.1111/exsy.12289

[51] Drury, B., Torgo, L. & Almeida, J. Classifying news stories to estimate the direction of a stock market index. In *6th Iberian Conference on Information Systems and Technologies (CISTI 2011)*, 1–4 (IEEE, 2011).

[52] Ahmed, K., Ali, M., Khalid, S. & Kamran, M. Framework for urdu news headlines classification. *J. Appl. Comput. Sci. Math.* (2016).

[53] Pisner, D. A. & Schnyer, D. M. Support vector machine. In *Machine learning*, 101–121 (Elsevier, 2020).

[54] Ali, M. *et al.**Machine learning based psychotic behaviors prediction from facebook status updates* (Computers, Materials and Continua, 2022).

[55] ul Mustafa, F. *et al.* Prediction of user’s interest based on urdu tweets. In *2020 International Symposium on Recent Advances in Electrical Engineering & Computer Sciences (RAEE & CS)*, vol. 5, 1–6 (IEEE, 2020).

[56] Nabeel, Z., Mehmood, M., Baqir, A. & Amjad, A. Classifying emotions in roman urdu posts using machine learning. In *2021 Mohammad Ali Jinnah University International Conference on Computing (MAJICC)*, 1–7 (IEEE, 2021).

[57] Butt, U. M. *et al.* Machine learning based diabetes classification and prediction for healthcare applications. *J. Healthc. Eng.* **2021** (2021). 10.1155/2021/9930985 PMC8500744 34631003

[58] Ali, M., Baqir, A., Psaila, G. & Malik, S. Towards the discovery of influencers to follow in micro-blogs (twitter) by detecting topics in posted messages (tweets). *Appl. Sci.* **10**, 5715 (2020). 10.3390/app10165715

[59] Ali, M., Mushtaq, H., Rasheed, M. B., Baqir, A. & Alquthami, T. Mining software architecture knowledge: Classifying stack overflow posts using machine learning. *Concurr. Comput. Pract. Exp.* **33**, e6277 (2021). 10.1002/cpe.6277

[60] Dilrukshi, I., De Zoysa, K. & Caldera, A. Twitter news classification using svm. In *2013 8th International Conference on Computer Science & Education*, pp. 287–291 (IEEE, 2013).

[61] Khan, T., Sherazi, H. H. R., Ali, M., Letchmunan, S. & Butt, U. M. Deep learning-based growth prediction system: A use case of china agriculture. *Agronomy* **11**, 1551 (2021). 10.3390/agronomy11081551

[62] Ho, T. K. Random decision forests. In *Proceedings of 3rd international conference on document analysis and recognition*, Vol. 1, 278–282 (IEEE, 1995).

[63] Ali, M., Scandurra, P., Moretti, F. & Sherazi, H. H. R. Anomaly detection in public street lighting data using unsupervised clustering. *IEEE Trans. Consum. Electron.* **70**, 4524–4535. (IEEE, 2024).

[64] Zhang, M.-L. & Zhou, Z.-H. A review on multi-label learning algorithms. *IEEE Trans. Knowl. Data Eng.* **26**, 1819–1837 (2013). 10.1109/TKDE.2013.39

